# Supplementary material for: Heat adaptation of phage T7 under an extended genetic code
Source: Virus Evol. 2021 Dec 1;7(2):veab100. doi: 10.1093/ve/veab100 (PMC8923235; doi:10.1093/ve/veab100)
Supplement: veab100_Supp [file veab100_supp.zip › Sequencing_Analysis.zip!/Sequencing_Analysis/A1/evidence/JC_SIDE_2_66.html]

BRESEQ :: Evidence


| New junction evidence | | | | | | | | | | | |
| --- | --- | --- | --- | --- | --- | --- | --- | --- | --- | --- | --- |
|  | | seq id | position | reads (cov) | reads (cov) | score | skew | freq | annotation | gene | product |
| \* | ? | NC\_001604.1 | = 36882 | 3450 (1.120) | 16 (0.010) | 15/582 | 37.4 | 0.48% | intergenic (–/–) | *–/–* | –/– |
|  | ? | NC\_001604.1 | 37265 = | 3289 (1.100) |  |  |  |  | intergenic (–/–) | *–/–* | –/– |
| Rejected: Coverage evenness skew score above cutoff. | | | | | | | | | | | |
| Rejected: Frequency below/above cutoff threshold. | | | | | | | | | | | |

Only 100 of 3403 total aligned reads displayed.  

`GCTATTCGGGTTAGGATGGCATCTAGGGTCAGACTCAATGGACGCTAAATGGAAACAGGAGGTACACAATGAGTACGTTAAGAGAGTTGAGGCTGCGAAGAGCACTCAAAGAGCAATCGATGCGGTATCTGCTAAGTATCAAGAAGACCTTGCCGCGCTGGAAGGGAGCACTGATAGGATTATTTCTGATTTGCGTAGCGACAATAAGCGGTTGCGCGTCAGAGTCAAAACTACCGGAACCTCCGATGGT‑CAGTGTGGATTCGAGCCTGATGGTCGAGCCGAACTTGACGACCGAGATGCTAAACGTATTCTCGCAGTGACCCAGAAGGGTGACGCATGGATTCGTGCGTTACAGGATACTATTCGTGAACTGCAACGTAAGTAGGAAATCAAGTAAGGAGGCAATGTGTCTACTCAATCCAATCGTAATGCGCTCGTAGTGGCGCAACTGAAAGGAGACTTCGTGGCGTTCCTATTCGTCTTATGGAAGGCGCTAAACCTACCGGTGCCCACTAAGTGTCAGATTGACATGGCTAAGGTGCTGGCGAATGGAGACAACAAGAAGTTCATCTTACAGGCTTTCCGTGGTATCGGTAAGTCGT  >  NC_001604.1/36964‑37565`  

                                                                                                                                                                                                                                                                                                              `|`                                                                                                                                                                                                                                                                                                              
`gCTATTCGGGTTAGGATGGCATCTAGGGTCAGACTCAATGGACGCTAAATGGAAACAGGAGGT‑‑ACAATGAGTACGTTAAGAGAGTTGAGGCTGCGAAGAGCACTCAAAGAGCAATCGATGCGGTATCTGATAAGTATCAAGAAGACCTTGCCGCGCTGGAAGGGAGCACTGATAGGATTATTTCTGATTTGCGTAGCGACAATAAGCGGTTGCGCGTCAGAGTCAAAACTACCGGAACCTCCGATGGT‑CAGTGTGGATTCGAGCCTGATGGTCGAGCCGAACTTGACGACCGAGATGCTaa                                                                                                                                                                                                                                                                                                             >  1:129708/1‑301 (MQ=255)`  
 `cTATTCGGGTTAGGATGGCATCTAGGGTCAGACTCAATGGACGCTAAATGGAAACAGGAGGT‑‑ACAATGAGTACGTTAAGAGAGTTGAGGCTGCGAAGAGCACTCAAAGAGCAATCGATGCGGTATCTGCTAAGTATCAAGAAGACCTTGCCGCGCTGGAAGGGAGCACTGATAGGATTATTTCTGATTTGCGTAGCGACAATAAGCGGTTGCGCGTCAGAGTCAAAACTACCGGAACCTCCGATGGT‑CAGTGTGGATTCGAGCCTGATGGTCGAGCCGAACTTGACGACCGAGATGCTaaa                                                                                                                                                                                                                                                                                                            >  1:218488/1‑301 (MQ=255)`  
 `cTATTCGGGTTAGGATGGCATCTACGGTCAGACTCAATGGACGCTAAATGGAAACAGGAGGT‑‑ACAATGAGTACGTTAAGAGAGTTGAGGCTGCGAAGAGCACTCAAAGAGCAATCGATGCGGTATCTGCTAAGTATCAAGAAGACCTTGCCGCGCTGGAAGGGAGCACTGATAGGATTATTTCTGATTTGCGTAGCGACAATAAGCGGTTGCGCGTCAGAGTCAAAACTACCGGAACCTCCGATGGT‑CAGTGTGGATTCGAGCCTGATGGTCGAGCCGAACTTGACGACCGAGATGCTaaa                                                                                                                                                                                                                                                                                                            >  1:307762/1‑301 (MQ=255)`  
  `tATTCGGGTTAGGATGGCAACTAGGGTCAGACAGAATGGACGCTAAATGAAAACAGGAGGTACA‑AA‑GAGTACGTTAAGGGAGTTGAGGCTGCGAAGAGCACTCAAAGAGCAATCGATGCGGTATCTGCTAAGTATCAAGAAGACCGTGCCGCGCTGAAAGGGAGCACTGATAGGAGTATTTCTGATTTGCGTAGCGACAATAAGCGGTTGCGCGTCAGAGTCAAAACTACCGGAACCTCCGATGGG‑CAGTGTGGATTCGAGCCTGATGGTCGAGCCGAACTTGACGACCGAGATGCTAAAc                                                                                                                                                                                                                                                                                                           <  1:369617/301‑1 (MQ=255)`  
            `agatgggtataagagaaaggACTCAATGGACGCTAAATGGAAACAGGAGGT‑‑ACAATGAGTACGTTAAGAGAGTTGAGGCTGCGAAGAGCACTCAAAGAGCAATAGATGCGGTATCTGCTAAGTATCAAGAAGACCTTGCCGCGCTGGAAGGGAGCACTGATAGGATTATTTCTGATTTGCGTAGCGAAAATAAGCGGTTGCGCGTCAGAGTCAAAACTACCGGAACCTCCGATGGT‑CAGTGTGGATTCGAGCCGGATGGTCGAGCCGAACTTGACGACCGAGATGCTAAACGTATTCTCGc                                                                                                                                                                                                                                                                                                 <  1:70024/282‑1 (MQ=255)`  
              `gATGGCATCTAGGGTCAGACTCAATGTACGCTAAATGGAAACAGGAGGTACACAATGAGTACGTTAAGAGAGTTGAGGCTGCGAAGAGCACTCAAAGAGCAATCGATGCGGTATCTGCTAAGTATCAAGAAGACCTTGCCGCGCTGGAAGGGAGCACTGATAGGATTATTTCTGATTTGCGTAGCGACAATAAGCGGTTGCGCGTCAGAGTCAAAACTACCGGAACCTCCGATGGT‑CAGTGTGGATTCGAGCCTGATGGTCGAGCCGAACTTGACGACCGAGATGCTAAACGTATTCTCGc                                                                                                                                                                                                                                                                                                 <  1:385388/301‑1 (MQ=255)`  
              `atgtgtataagagacaggACTCAATGGACGCTAAATGGAAACAGGAGGT‑‑ACAATGAGTACGTTAAGAGAGTTGAGGCTGCGAAGAGCACTCAAAGAGCAATCGATGCGGTATCTGCTAAGTATCAAGAAGACCTTGCCGCGCTGGAAGGGAGCACTGATAGGATTATTTCTGATTTGCGTAGCGACAATAAGCGGTTGCGCGTCAGAGTCAAAACTACCGGAACCTCCGATGGT‑CAGTGTGGATTCGAGCCTGATGGTCGAGCCGAACTTGACGACCGAGATGCTAAACGTATTCTCGCAg                                                                                                                                                                                                                                                                                               <  1:256225/284‑1 (MQ=255)`  
                       `aaGGGTATGAATCAATGGACGCTAAATGGAAACAGGAGGT‑‑ACAATGAGAACGTTAAGAGAGTGGAGGCTGCGAAGAGCACTGAAAGAGCAATCGATGCGGTATCTGCTAAGTATCAAGAAGACCTTGCCGCGCTGGAAGGGAGCACGGATAGGATTATTTCTGATTTGCGTAGCGACAATAAGCGGTTGCGCGTCAGAGTCAAAACTACCGGAACCTCCGATGGT‑CAGTGTGGATTCGAGCCTGATGGTCGAGCCGAACTTGACGACCGAGATGCTAAACGTATTCTCGCAGTGACCCAGa                                                                                                                                                                                                                                                                                      <  1:296211/300‑1 (MQ=255)`  
                        `aGGGTCAGACTCAATGGACGCTAAATGGAAACAGGAGGT‑‑ACAATGAGTACGTTAAGAGAGTTGAGGCTGCGAAGAGCACTCAAAGAGCAATCGATGCGGTATCTGCTAAGTATCAAGAAGACCTTGCCGCGCTGGAAGGGAGCACTGATAGGATTATTTCTGATTTGCGTAGCGACAATAAGCGGTTGCGCGTCATAGTCAAAACTACCGGAACCTTCGATTGT‑CAGTGTGGATTAGAGCCTGATGGTCGAGCAGAACTTGACGACCGAGATGCTAAACGTATTCTCGCAGTGACCCAgca                                                                                                                                                                                                                                                                                     >  1:199614/1‑299 (MQ=255)`  
                         `gggTCAGACTCAATGGACGCTAAATGGAAACAGGAGGT‑‑ACAATGAGTACGTTAAGAGAGTTGAGGCTGCGAAGAGCACTCAAAGAGCAATCGATGCGGTATCTGCTAAGTATCAAGAAGACCTTGCCGCGCTGGAAGGGAGCACTGATAGGATTATTTCTGATTTGCGTAGCGACAATAAGCGGTTGCGCGTCAGAGTCAAAACTACCGGAACCTCCGATGGT‑CAGTGTGGATTCGAGCCTGATGGTCGAGCCGAACTTGACGACCGAGATGCTAAACGTATTCTCGCAGTGACCCAGAAg                                                                                                                                                                                                                                                                                    >  1:481803/1‑301 (MQ=255)`  
                             `aaGAATCAATGGACGCTAAATGGAAACAGGAGGTACACAATGAGTACGTTAAGAGAGTTGAGGCTGCGAAGAGCACTCAAAGAGCAATCGATGCGGTATCTGCTAAGTATCAAGAAGACCTTGCCGCGCTGGAAGGGAGCACTGATAGGATTATTTCTGATTTGCGTAGCGACAATAAGCGGTTGCGCGTCAGAGTCAAAACTACCGGAACCTCCGATGGT‑CAGTGTGGATTCGAGCCTGATGGTCGAGCCGAACTTGACGACCGAGATGCTAAACGTATTCTCGCAGTGACCCAGAAggg                                                                                                                                                                                                                                                                                  <  1:10720/300‑1 (MQ=255)`  
                               `gACTCAATGGACGCTAAATGGAAACAGGAGGT‑‑ACAATGAGTACGTTAAGAGAGTTGAGGCTGCGAAGAGCACTCAAAGAGCAATCGATGCGGTATCTGCTAAGTATCAAGAAGACCTTGCCGCGCTGGAAGGGAGCACTGATAGGATTATTTCTGATTTGCGTAGCGACAATAAGCGGTTGCGCGTCAGAGTCAAAACTACCGGAACCTCCGATGGT‑CAGTGTGGATTCGAGCCTGATGGTCGAGCCGAACTTGACGACCGAGATGCTAAACGTATTCTCGCAGTGCCCCAGAAgctgtct                                                                                                                                                                                                                                                                              >  1:185075/1‑295 (MQ=255)`  
                                 `cTCAATGGCCGCTAAATGGAAACAGGAGGT‑‑ACAATGAGTACGTTAAGAGAGTTGAGGCTGCGAAGAGCACTCAAAGAGCAATCGATGCGGTATCTGCTAAGTATCAAGAAGACCTTGCCGCGCTGGAAGGGAGCACTGATAGGATTATTTCTGATTTGCGTAGCGACAATAAGCGGTTGCGCGTCAGAGTCAAAACTACCGGAACCTCCGATGGT‑CAGTGTGGATTCGAGCCTGATGGTCGAGCCGAACTTGACGACCGAGATGCTAAACGTATTCTCGCAGTGACCCAGAAgctgtctct                                                                                                                                                                                                                                                                            >  1:208186/1‑293 (MQ=255)`  
                                             `gtataagagaCAGGAGGT‑‑ACAATGAGTACGTTAAGAGAGTTGAGGCTGCGAAGAGCACTCAAAGAGCAATCGATGCGGTATCTGCTAAGTATCAAGAAGACCTTGCCGCGCTGGAAGGGAGCACTGATAGGATTATTTCTGATTTGCGTAGCGACAATAAGCGGTTGCGCGTCAGAGTCAAAACTACCGGAACCTACGATGGT‑CAGTGTGGATTCGAGCCTGATGGTCGAGCCGAACTTGACGACCGAGATGCTAAACGTATTCTCGCAGTGACCCAGAAGGGTGACGCATGGATTCGTGc                                                                                                                                                                                                                                                                <  1:399212/292‑1 (MQ=255)`  
                                              `aaaTGGAAACAGGAGGT‑‑ACAATGAGTACGTTAAGAGAGTTGAGGCTGCGAAGAGCACTCAAAGAGCAATCGATGCGGTATCTGCTAAGTATCAAGAAGACCTTGCCGCGCTGGAAGGGAGCACTGATAGGATTATTTCTGATTTGCGTAGCGACAATAAGCGGTTGCGCGTCAGAGTCAAAACTACCGGAACCTCAGATGGT‑CAGTGTGGATTCGAGCCTGATGGTCGAGCCGAACTTGACGACCGAGATGCTAAACGTATTCTCGCAGTGACCCAGAAGGGTGACGCactgtctcttata                                                                                                                                                                                                                                                               >  1:375089/1‑289 (MQ=255)`  
                                                  `ggAAACAGGAGGT‑‑ACAATGAGTACGTGAAGAGAGTTGAGGCTGCGAAGAGCACTCAAAGAGCAATCGATGCGGTATCTGCTAAGTATCAAGAAGACCTTGCCGCGCTGGAAGGGAGCACTGATAGGATTATTTCTGATTTGCGTAGCGACAATAAGCGGCTGCGCGTCAGAGTCAAAACTACCGGAACCTCCGATGGT‑CAGTGTGGATTCGAGCATGATGGTCGAGCCGAACTTGACGACCGTGATGCTAAACGTATTCTCGCAGTGACCCAGAAGGGTGACGCATGGATTCGTGCGTTaa                                                                                                                                                                                                                                                           >  1:193382/1‑300 (MQ=255)`  
                                                  `gaaaaCAGGAGGT‑‑ACAATGAGTACGTGAAGAGAGTTGAGGCTGCGAAGAGCACTCAAAGAGCAATCGATGCGGTATCTGCTAAGTATCAAGAAGACCTTGCCGCGCTGGAAGGGAGCACTGATAGGATTATTTCTGATTTGCGTAGCGACAATAAGCGGTTGCGCGTCAGAGTCAAAACTACCGGAACCTCCGATGGT‑CAGTGTGGATTCGAGCCTGATGGTCGAGCCGAACTTGACGACCGAGATGCTAAACGTATTCTCGCAGTGACCCAGAAGGGTGACGCATGGATTCGTGCGTTAc                                                                                                                                                                                                                                                           <  1:278111/299‑1 (MQ=255)`  
                                                    `aaaCAGGAGGT‑‑ACAATGAGTACGTTAAGAGATTTGACGCTGCGAAGAGCACTCAAAGAGCAATTGTTGCGGTACCTGCTAAGTATCAAGCAGACCTTGCCGCGCTGGAAGGGAGCACTTATAGGATTATTTCTGATTTGCGTAGCGACAATAAGCGGTTGCGCGTCAGAGACATAACTACCGGAACCCCCGATGGT‑CAGTGTGGATTCGAGCCTGATGGTCGAGCCGAACTTGACGACCGAGATGCTAAACGTATTCTCGCAGTGACCCGGAAGGGTGACGCATGGATTCGTGCGTTACAg                                                                                                                                                                                                                                                         <  1:95828/301‑1 (MQ=255)`  
                                                    `aaaCAGGAGGT‑‑ACAATGAGGACGTTAAGGGTGTTGAGGCTGCGAAGAGCACTCAAAGAGCAATCGATGCGGTATCTTCTAAGTATCAAGAAGACCTTGCCGCGCTGGAAGGGAGCACTGATAGGATTATTTCTGATTTGCGTAGCGACAATAAGCGGTTGCGCGTCAGAGTCAAAACTACCGGAACCTCCGATGGT‑CAGTGTGGATTCGAGCCTGATGGTCGAGCCGAACTTGACGACCGAGATGCTAAACGTATTCTCGCAGTGACCCAGAAGGGTGACGCATGGATTCGTGCGTTACAg                                                                                                                                                                                                                                                         <  1:111404/301‑1 (MQ=255)`  
                                                      `ggctcggagatgtggataagagacagagagagTTGAGGCTGCGAAGAGCACTCAAAGAGCAATCGATGCGGTATCTGCTAAGTATCAAGAAGACCTTGCCGCGCTGGAAGGGAGCACTGATAGGATTATTTCTGATTTGCGTAGCGACAATAAGCGGTTGCGCGTCAGAGTCAAAACTACCGGAACCTCCGATGGT‑CAGTGTGGATTCGAGCCTGATGGTCGAGCCGAACTTGACGACCGAGATGCTAAACGTATTCTCGCAGTGACCCAGAAGGGTGACGCATGGATTCGTGCGTTACAg                                                                                                                                                                                                                                                         <  1:180485/275‑1 (MQ=255)`  
                                                      `aCAGGAGGT‑‑ACAATGAGTACGTTAAGAGAGTTGAGGCTGCGAAGAGCACTCAAAGAGCAATCGATGCGGTATCTGCTAAGTATCAAGAAGACCTTGCCGCGCTGTAAGGGAGCACTGATAGGATTATTTCTGATTTGCGTAGCGACAATAAGCGGTTGCGCGTCAGAGTCAAAACTACCGGAACCTCCTATGGT‑CAGTGTGGATTCGAGCCTGATGGTCGAGCCGAACTTGACGACCGAGATGCTAAACGTATTCTCGCAGTGACCCCGAAGGGTGACGCATGTATTCGTGCGTTACAGGa                                                                                                                                                                                                                                                       >  1:161167/1‑301 (MQ=255)`  
                                                       `cAGGAGGT‑‑ACAATGAGTACGTTAAGAGAGTTGAGGCTGCGAAGAGCACTCAAAGAGCAATCGATGCTGTATCTGCTAAGTATCAAGAAGACCTTGCCGCGCTGGAAGGGAGCACTGATAGGATTATTTCTGATTTGCGTAGCGACAATAAGCGGTTGCGCGTCAGAGTCAAAACTACCGGAACCTCCGATGGT‑CAGTGTGGATTCGAGCCTGATGGTCGATCCGAACTTGACGACCGAGATGCTAAACGTATTCTCGCAGTGACCAAGAAGGGTGACGCATGGATTCGTGCGTTACAGGAt                                                                                                                                                                                                                                                      <  1:91728/301‑1 (MQ=255)`  
                                                           `ggaggtacaATGAGTACGTTAAGAGAGTTGAGGCTGCGAAGAGCACTCAAAGAGCAATCGATGCGGTATCTGCTAAGTATCAAGAAGACCTTGCCGCGCTGGAAGGGAGCACTGATAGGATTATTTCTGATTTGCGTAGCGACAATAAGCGGTTGCGCGTCAGAGTCAAAACTACCGGAACCTCCGATGGT‑CAGTGTGGATTCGAGCCTGATGGTCGAGCCGAACTTGACGACCGAGATGCTAAACGTATTCTCGCAGTGACCCAGAAGGGTGACGCATGGATTCGTGCGTTACAGGATAc                                                                                                                                                                                                                                                    <  1:79789/295‑1 (MQ=255)`  
                                                           `ggaggtacaATGAGTACGTTAAGAGAGTTGAGGCTGCGAAGAGCACTCAAAGAGCAATCGATGCGGTATCTGCTAAGTATCAAGAAGACATTGCCGCGCTGGAAGGGAGCACTGATAGGATTATTTCTGATTTGCGTAGCGACAATAAGCGGTTGCGCGTCAGAGTCAAAACTAACGGAACCTCCGATGGT‑CAGTGTGGATTCGAGCCTGATGGTCGAGCCGAACTTGACGACCGAGATGCTGAACGTATTATCGCAGTGAACCAGAAGGGTGACGCATGGATTAGTGCGGTACAGGaaaa                                                                                                                                                                                                                                                    >  1:292066/7‑298 (MQ=255)`  
                                                           `aagggtacaATGAGGACGTTAAGAGAGTTGAGGCTTCGAAGAGCACTCAAAGAGAAATCGATGCGGTATCTGCTAAGTATCAAGAAGACCTTGCCTCGCTGGAAGGGAGCACTGATAGGATTATTTCTGATTTGCGTAGCTACAATAAGCGGTTGCGCGTCAGAGTCAAAACTACCGGAACCTCCGATGGT‑CAGTGTGGATTCGAGCCTGATGGTCGAGCCGAACTTGACGACCGAGATGCTAAACGTATTCTCGCAGTGACCCAGAAGGGTGACGCATGGATTCGTGCGTTACAGGATAc                                                                                                                                                                                                                                                    <  1:300148/295‑1 (MQ=255)`  
                                                               `gtacaATGAGTACGTTAAGAGAGTTGAGGCTGCGAAGAGCACTCAAAGAGCAATCGATGCGGTATCTGCTAAGTATCAAGAAGACCTTGCCGCGCTGGAAGGGAGCACTGATAGGATTATTTCTGATTTGCGTAGCGACAATAAGCGGTTGCGCGTCAGAGTCAAAACTACCGGAACCTCCGATGGT‑CAGTGTGGATTCGAGCCTGATGGTCGAGCCGAACTTGACGACCGAGATGCTAAACGTATTCTCGCAGTGACCCAGAAGGGTGACGCATGGATTCGTGCGTTACAGGATACTAtc                                                                                                                                                                                                                                                >  1:338674/3‑300 (MQ=255)`  
                                                               `gtacaATGAGTACGTTAAGAGAGTTGAGGCTGCGAAGAGCACTCAAAGAGCAATCGATGCGGTATCTGCTAAGTATCAAGAAGACCTTGCCGCGCTGGAAGGGAGCACTGATAGGATTATTTCTGATTTGCGTAGCGACAATAAGCGGTTGCGCGTCAGAGTCAAAACTACCGGAACCTCCGATGGT‑CAGTGTGGATTCGAGCCTGATGGTCGAGCCGAACTTGACGACCGAGATGCTAAACGTATTCTCGCAGTGACCCAGAAGGGAGACGCATGGATTCGTGCGTTACAGGAGAcctgt                                                                                                                                                                                                                                                >  1:506793/3‑297 (MQ=255)`  
                                                               `gtacaATGAGTACGTTAAGAGAGTTGAGGCTGCGAAGAGCACTCAAAGAGCAATCGATGCGGTATCTGCTAAGTATCAAGAAGACCTTGCCGCGCTGGAAGGGAGCACTGATAGGATTATTTCTGATTTGCGTAGCGACAATAAGCGGTTGCGCGTCAGAGTCAAAACTACCGGAACCTCCGATGGT‑CAGTGTGGATTCGAGCCTGATGGTCGAGCCGAACTTGACGACCGAGATGCTAAACGTATTCTCGCAGTGACACAGAAGGGTGACGCATGGATTCGTGCGTTACAGGATACTAtt                                                                                                                                                                                                                                                >  1:500698/3‑301 (MQ=255)`  
                                                                   `agagacagcGTGAAGAGAGTTGAGGCTGCGAAGAGCACTCAAAGAGCAATCGATGCGGTATCTGCTAAGTATCAAGAAGCCCTTGCCGCGCTGGAAGGGAGCACTGATAGGATTATTTCTGATTTGCGTAGCGAAAATAAGCGGTTGCGCGTCAGAGTCAAAACTACCGGAACCTCCGATGGT‑CAGTGTGGATTCGAGCCTGATGGTCGAGCCGAACTTGACGACCGAGATGCTAAACGTATTCTCGCAGTGACCCAGAAGGGTGACGCATGGATTCGTGCGTTACAGGATACTATTCGTg                                                                                                                                                                                                                                            <  1:473176/293‑1 (MQ=255)`  
                                                                      `gAGTACGTTAAGAGAGTTGAGGCTGCGAAGAGTACTCAAAGAGCAATCGATGCGGTATCTGCTAAGTATCAAGAAGACCTTGCCGCGCTGGAAGGGAGCACTGATAGGATTATTTCTGATTTGCGTAGCGACAATAAGCGGTTGCGCGTCAGAGTCAAAACTACCGGAACCTCCGATGGT‑CAGTGTGGCTTCGAGCCTGATGGTCGAGCCGAACTTGACGACCGAGATGCTAAACGTATTCTCGCAGTGACCCAGAAGGGTGACGCATGGCATCGTTCGTTACAGGATACTATTCGTGAAc                                                                                                                                                                                                                                         >  1:281243/1‑301 (MQ=255)`  
                                                                         `tACGTTAAGAGAGTTGAGGCTGCGAAGAGCACTCAAAGAGCAATCGATGCGGTATCTGCTAAGTATCAAGAAGACCGTGCCGCGCTGGAAGGGAGCACTGATAGGATTATTTCTGATTTGCGTAGCGACAATAAGCGGTTGCGCGTCAGAGTCAAAACTACCGGAACCTCCGATGGT‑CAGTGTGGATTCGAGCCTGATGGTCGAGCCGAACTTGACGACCGAGATGCTAAACGTATTCTCGCAGTGACCCAGAAGGGTGACGCATGGATTCGTGCGTTACAGGATACTATTCGTGAACTGc                                                                                                                                                                                                                                      <  1:352888/301‑1 (MQ=255)`  
                                                                                      `aagAGGATGCGAAAAGCACTGAAATAAGAAGAGATGCGGTAACGTCTAAGGAACAAGAAGACCTAGGCGCGCTGGAAGGGGGCACTGATAGGATTATTTCTAATTTGCGTAGCGACAATAAGCGGTTGCGCGTGAGAGGCAAAACTACCGGAACCTGAGATGGT‑AAGAGTGGATTCGAGCCTGAAGGTCGAGCCGAACTTGACGACCGAGATGCTAAACGTATTATCGCAGTGACCCAGAAGGGTGACGCATGGATTCGTGCGTTACAGGATACTATTCGTGAACTGCAACGTAAGTAGGa                                                                                                                                                                                                                         <  1:260338/299‑1 (MQ=255)`  
                                                                                          `ggCTGCGAATAGCACTCAAAGAGCAATCGATGCGGTATCTGCTAAGTATCAAGAATACCTTGCCGCGCTGGAAGGGAGCACTGATAGGATTATTTCTGATTTGCGTAGCGACAATAAGCGGTTGCGCGTCAGAGTCAAAACTACCGGAACCTCCGATGGT‑CAGTGTGGATTCGAGCCTGATGGTCGAGCCGAACTTGACTACCGAGATGCTAAACGTATTCTCGCAGTGACCCAGAAGGGTGACGCATGGATTCGTGCGTTACAGGATACTATTCGTGAACTGCAACGTAAGCAGGAAATc                                                                                                                                                                                                                     <  1:269490/301‑1 (MQ=255)`  
                                                                                             `agCGAAGAGAACTCAAAGAGCAATCGATGAGGTATCTGCTAAGTAACAAGAAGACCTGGCCGCGCTGGAAGGGAGGACTGATAGGATTATGTCTGATTTGCGTGGCGACAATAAGCGGTTGCGCGTCAGAGTCAAAACTACCGGAACCTCCGATGGT‑CAGTGTGGATTCGAGCCTGATGGTCGAGCCGAACTTGACGACCGAGATGCTAAACGTATTCTCGCAGTGACCCAGAAGGGTGACGCATGGATTCGTGCGTTACAGGATACTATTCGTGAACTGCAACGTAAGCAGGAAATCAAg                                                                                                                                                                                                                  <  1:342970/300‑1 (MQ=255)`  
                                                                                              `gCGAAGAGCACTCAAAGAGCAATCGATGCGGTATCTGCTAAGTATCAAGAAGACCTTGCCGCGCTGGAAGGGAGCACTGATAGGATTATTTCTGATTTGCGTAGCGACAATAAGCGGTTGCGCGGCAGAGTCAAAACTACCGGTACCTCCGATGGT‑CAGTGTGTATTCGAGCCTGATGGTCGAGGCGAACTTGACGACCGAGATGCTAAACGTATTCTCGCAGTGACCAAGAAGGGTGACGCATGGATACGTTCGTTACAGGCTACTATTAGTGAACTGGAAAGTACGTAGGAAAGCAAga                                                                                                                                                                                                                 >  1:490767/1‑300 (MQ=255)`  
                                                                                                `gAAGAGCACTCAAAGAGCAATCGATGCGGTATCTGCTAAGTATCAAGAAGACCTTGCCGCGCTGGAAGATAGCACTGATAGGATTATTTCTGATTTGCGTAGCGACAATAAGCGGCTGCGCGTCAGAGTCAAAACTACCGGAACCTCCGATGGT‑CAGTGTGGATTCGAGCCTGATGGTCGAGCCGAACTTGACGACCGTGATGCTAAACGTATTCCCGCAGTGACCCACAAGGGTGACGCATCGATTCTTGCGTTACAGGATACTCTTCGTGAACTGCAACGTAAGTAGGAAATCAAGtct                                                                                                                                                                                                               >  1:3312/1‑299 (MQ=255)`  
                                                                                                  `agagCACTCAAAGAGCAATCGATGCGGTATCTGCTAAGTATCAAGAAGACCTTGCCGCGCTGGAAGGGAGCACTGATAGGATTATTTCTGATTTGCGTAGCGACAATAAGCGGTTGCGCGTCAGAGTCAAAACTACCGGAACCTCCGATGGT‑CAGTGTGGATTCGAGCATGATGGTCGAGCCGAACTTGACGACCGAGATGCTAAACGTATTCTCGCAGTGACCCAGAAGGGTGACGCATGGATTCGTGCGTTACCGGATACTATTCGTTAACTGCAACGTAAGCAGGAATTCAAGTAagg                                                                                                                                                                                                             >  1:334902/1‑301 (MQ=255)`  
                                                                                                   `gagCACTCAAAGAGCAATCGATGCGGTATCTGCTAAGTATCAAGAAGACCTTGCCGCGCTGGAAGGGAGCACTGATAGGATTATTTCTGATTTGCGTAGCGACAATAAGCGGTTGCGCGTCAGAGTCAAAACTACCGGAACCTCCGATGGT‑CAGTGTGGATTCGATCCTGATGGTCGAGCCGAACTTGACGACCGAGATGCTAAACGTATTCTCGCAGTGACCCAGAAGGGTGACGCATGGATTCGTGCGTTACAGGATACTATTCGTGAACTGCAACGTAAGTAGGAAATCAAGTAagta                                                                                                                                                                                                            >  1:425194/1‑299 (MQ=255)`  
                                                                                                        `cTCAAAGAGCAATCGATGCGGTATCTGCTAAGTATCAAGAAGACCTTGCCGCGCTGGAAGGGAGCACTGATAGGATTATTTCTGATTTGCGTAGCGACAATAAGCGGTTGCGCGTCAGAGTCAAAACTACCGGAACCTCCGATGGT‑CAGTGTGGATTCGAGCCTGATGGTCGAGCCGAACTTGACGACCGAGATGCTAAACGTATTCTCGCAGTGACCCCGAAGGGTGACGCATGGATTCGTGCGTTACAGGATACTCTTCGTGAACTGCAACGTAAGCAGGAAATCAAGTaactgtcgct                                                                                                                                                                                                       >  1:65142/1‑293 (MQ=255)`  
                                                                                                        `cTCAAAGAGCAATCGATGCGGTATCTGCTAAGTATCAAGAAGACCTTGCCGCGCTGGAAGGGAGCACTGATAGGATTATTTCTGATTTGCGTAGCGACAATAAGCGGTTGCGCGTCAGAGTCAAAACTACCGGAACCTCCGATGGT‑CAGTGTGGATTCGAGCCTGATGGTCGAGCCGAACTTGACGACCGAGATGCTAAACGTATTCTCGCAGTGACCCAGAAGGGTGACGCATGGATTCGTGCGTTACAGGATACTATTCGTGAACTGCAACGTAAGTAGGAAATCAAGTAAGGAGGCaa                                                                                                                                                                                                       >  1:311801/1‑301 (MQ=255)`  
                                                                                                          `aaaaGAGCAATCGATGCGGTATCTGCTAAGTATCAAGAAGACCTTGCCGCGCTGGAAGGGAGCACTGATAGGATTATTTCTGATTTGCGTAGCGACAATAAGCGGTTGCGCGTCAGAGTCAAAACTACCGGAACCTCCGATGGT‑CAGTGTGGATTCGAGCCTGATGGTCGAGCCGAACTTGACGACCGAGATGCTAAACGTATTCTCGCAGTGACCCAGAAGGATGACGCATGGATTCGTGCGTTACAGGATACTATTCGTGAACTGCAACGTAAGTAGGAAATCAAGTAAGGAGACAAtg                                                                                                                                                                                                     <  1:364639/300‑1 (MQ=255)`  
                                                                                                              `gagCAATCGATGCGGTATCTGCTAAGTATCAAGAAGACCTTGCCGCGCTGGAAGGGAGCACTGATAGGATTATTTCTGATTTGCGTAGCGACAATAATCGGTTGCGCGTCAGAGTCAAAACTACCGGAACCTCCGATGGT‑CAGTGTGGATTCGAGCCTGATGGTCGAGCCGAACTTGACGACCGAGATGCTAAACGTATTCTCGCAGTGACCCAGAAGGGTGACGCATGGATTCGTGCGTTACAGGATACTATTCGTGAACTGCAACGTAAGTAGGAAATCAAGTAAGGAGGCAATGTGTc                                                                                                                                                                                                 <  1:348694/301‑1 (MQ=255)`  
                                                                                                                 `taaTCGATGCGGTATGTTCTAACCATCAAAAAGACCTTGCCGCGCTCGGAGGGAGCACTGATAGGATTATTTCTGATTTGCGTGGCGACAATAAGCGGTTGCGCGTCAGAGTCAAAACTACCGGAACCTCCGATGGT‑CAGTGTGGATTCGAGCCTGATGGTCGAGCCGAACTTGACGACCGAGATGCTAAACGTATTCTCGCAGTGACCCAGAAGGGTGACGCATGGATTCGTGCGTTACAGGATACTATTCGTGAACTGCAACGTAAGTAGGAAATCAAGTAAGGAGGCAATGTGTCTAc                                                                                                                                                                                              <  1:100214/300‑1 (MQ=255)`  
                                                                                                                 `aaaTCGATGCGGGATCTGCTAAGTATCAAGAAGACCTTGCCGCGCTGGAAGGGAGCACTGATAGGATGATTTCTGATTTGCGTAGCGACAATAAGCGGTTGCGCGTCAGAGTCAAAACTACCGGAACCTCCGATGGT‑CAGTGTGGATTCGAGCCTGATGGTCGAGCCGAACTTGACGACCGAGATGCTAAACATATTCTCGCAGTGACCCAGAAGGGTGACGCATGGATTCGTGCGTTACAGGATACTATTCGTGAACTGCAACGTAAGTAGGAAATCAAGTAAGGAGGCAATGTGTCTAc                                                                                                                                                                                              <  1:156168/300‑1 (MQ=255)`  
                                                                                                                   `aTCGATGCGGTATCTGCTAAGTATCAAGAAGACCTTGCCGCGCTGGAAGGGAGCACTGATAGGATTATTTCTGATTTGCGTAGCGACAATAAGCGGTTGCGCGTCAGAGTCAAAACTACCGGAACCTCCGATGGT‑CAGTGTGGATTCGAGCCTGATGGTCGAGCCGAACTTGACGACCGAGATGCTAAACGTATTCTCGCAGTGACCCAGAAGGGTGACGCATGGATTCGTGCGTTACAGGATACTATTCGTGAACTGCAACGTAAGTAGGAAATCAAGTAAGGAGGCAATGTGTCTACTc                                                                                                                                                                                            <  1:147230/301‑1 (MQ=255)`  
                                                                                                                   `aTCGATGCGGTATCTGCTAAGTATCAAGAAGACATTGCCGAGCTGGAAGGGAGCACTGATAGGATTATTTCTGATTTGCGTAGCGACAATAAGCGGTTGCGCGTCAGAGTCAAAACTACCGGAACCTCCGATGGT‑CAGTGTGGATTCGAGCCTGATGGTCGAGCCGAACTTGACGACCGAGAAGCTAAACGTATTCTCGCAGTGACCCAGAAGGGTGACGCATGGATTCGTGCGTTACAGGATACTATTCGTGCACTGCAACGTAAGTAGGAAATCAAGTAAGGAGGCAATGTGTCTACTc                                                                                                                                                                                            >  1:212207/1‑301 (MQ=255)`  
                                                                                                                   `aTCAATGCGGTATCTGCTAAGTATCAAGAAGACCTGGCCGCGCTGGAAGGGAGAACTTATAGGATTATTTCTGATTTGCGTAGCGACAATAAGCGGTTGCGCGTCAGAGTCAAAACTACCGGAACCTCCGATGGT‑CAGTGTGGATTCGAGCCTGATGGTCGAGCCGAACTTGACGACCGAGATGCTAAACGTATTCTCGCAGTGACCCAGAAGGGTGACGCATGGATTCGTGCGTTACAGGATACTATTCGTGAACTGCAACGTAAGTAGGAAATCAAGTAAGGAGGCAATGTGTCTACTc                                                                                                                                                                                            <  1:40592/301‑1 (MQ=255)`  
                                                                                                                     `cGATGCGGTATCTGCTAAGTATCAAGAAGACCTTGCCGCGCTGGAAGGGAGCACTGATAGGATTATTTCTGATTTGCGTAGCTACAATAAGCGGTTGCGCGTCAGAGTCAAAACTACCGGAACCTCCGATGGT‑CAGTGTGGATTCGAGCCTGATGGTCGAGCCGAACTTGACTACCGAGATGCTAAACGTATTCTCGCAGTGACCCAGAAGGGTGACGCATGGATTCGTGCGTTACAGGATACTATTCGTGAACTGCAACGTAAGTAGGAAATCAAGTAAGGAGGCAATGTGTCTACTcaa                                                                                                                                                                                          >  1:496036/1‑301 (MQ=255)`  
                                                                                                                       `aTGCGGTATCTGCTAAGTATCAAGAAGACCTTGCCGCGCTGGAAGGGAGCACTGATAGGATTATTTCTGATTTGCGTAGCGACAATAAGCGGTTGCGCGTCAGAGTCAAAACTACCGGAACCTCCGATGGT‑CAGTGTGGATTCGAGCCTGATGGTCGGGCCGAACTTGACGACCGAGATGCTAAACGTATTCTCGCAGTGACCCAGAAGGGTGACGCATGGATTCGTGCGTTACAGGATACTATTCGTGAACTGCAACGTAAGTAGGAAATCAAGTAAGGAGGCAATGTGTCTACTcaatc                                                                                                                                                                                        >  1:380961/1‑301 (MQ=255)`  
                                                                                                                        `ggCGGTATCTGCTAAGTATCAAGAAGACCTTGCCGCGCTGGAAGGGAGCACTGATAGGATTATTTCTGATTTGCGTAGCGACAATAAGCGGTTGCGCGTCAGAGTCAAAACTACCGGAACCTCCGATGGT‑CAGTGTGGATTCGAGCCTGATGGTCGAGCCGAACTTGACGACCGAGATGCTAAACGTATTCTCGCAGTGACCCAGAAGGGTGACGCATGGATTCGTGCGTTACAGGATACTATTCGTGAACTGCAACGTAAGTAGGAAATCAAGTAAGGATGCAATGTGTCTACTcaatcc                                                                                                                                                                                       <  1:138539/300‑1 (MQ=255)`  
                                                                                                                        `aagggTATCTGCTAAGTATCAAGAAGACCTTGCCGCGCTGGAAGGGAGCACTGATAGGATTATTTCTGATTTGCGTAGCGAAAATAAGCGGTGGCGCGTCAGAGTCAAAACTACCGGAACCTCCGATGGT‑CAGTGTGGATTAGAGCCTGATGGTCGAGCCGAACTTGACGACCGAGATGCTAAACGTATTCTCGCAGTGACCCAGAAGGGTGACGCATGGATTCGTGCGTTACAGGATACTATTCGTGAACTGCAACGTAAGTAGGAAATCAAGTAAGGAGGCAATGTGTCTACTcaatcc                                                                                                                                                                                       <  1:389135/298‑1 (MQ=255)`  
                                                                                                                           `gcagtcgacTAAGTATCAAGAAGACATTGTCGCGATGGAAGGGAGCACTGATAGGATTATTGATGATTTGCGTCGCAACAATAAGCGGGTGCGCGTCAGAGTCAAAACTACCGGAACCTCCGATGGT‑TAGTGTGGATTCGAGCCTGATGGTCGAGCCGAACTTGACGACCGAGATGCTAAACGTATTCTAGCAGTGACCCAGAAGGGTGACGCATGGATTCGTGCGTTACAGGATACTATTCGGGAACTGCAACGTAAGTAGGAAATCAAGTAAGGAGGCAATGTGTCTACTaaatccaat                                                                                                                                                                                    <  1:303316/293‑1 (MQ=255)`  
                                                                                                                            `gTATCTGCTAAGTATCAAGAAGACCTTGCCGCGCTGGAAGGGAGCACTGATAGGATTATTTCTGATTTGCGTAGCGACAATAAGCGGTTGCGCGTCAGAGTCAAAACTACCGGAACCTCCGATGGT‑CAGTGTGGATTCGAGCCTGATGGTCGAGCCGAACTTGACGACCGAGATGCTAAACGTATTCTCGCAGTGACCCAGAAGGGTGACGCATGGATTCGTGCGTTACAGGATACTATTCGTGAACTGCAACGTAAGTAGGAAATCAAGTAAGGAGGCAATGTGTCTAcctgtctcttat                                                                                                                                                                                   >  1:463037/1‑290 (MQ=255)`  
                                                                                                                             `tATCTGCTAAGTATCAAGAAGACCTTGCCGCGCTGGAAGGGAGCACTGATAGGATTATTGCTGATTTGCGTAGCGACAATAAGCGGTTGCGCGTCAGAGTCAAAACTACCGGAACCTCCGATGGT‑CAGTGTGGATTCGAGCCTGATGGTCGAGCCGAACTTGACGACCGAGATGCTAAACGTATTCTCGCAGTGACCCAGAAGGGTGACGCATGGATTCGTGCGTTACAGGATACTATTCGTGAACTGCAACGTAAGTAGGAAATCAAGTAAGGAGGCAATGTGTCTACTCAATCCAATCg                                                                                                                                                                                  <  1:88438/301‑1 (MQ=255)`  
                                                                                                                              `aTCTGCTAAGTATCAAGAAGACCTTGCCGCGCTGGAAGGGAGCACTGATAGGATTATTTCTGATTTGCGTAGCGACAATAAGCGGTTGCGCGTCAGAGTCAAAACTACCGGAACCTCCGATGGT‑CAGTGTGGATTCGAGCCTGATGGTCGAGCCGAACTTGACGACCGAGATGCTAAACGTATTCTCGCAGTGACCCAGAAGGGTGACGCCTGGATTCGTGCGTTACAGGATACTATTCGTGAACTGCAACGTAAGTAGGAAATCAAGTAAGGAGGCAATGTGTCTACTCAATCCAATCGt                                                                                                                                                                                 >  1:55863/1‑301 (MQ=255)`  
                                                                                                                                `cTGCTAAGTATCAAGAAGACCTTGCCGCGCTGGAATGGAGCACTGATAGGATTATTTCTGATTTGCGTATCGACAATAAGCGGTTGCGCGTCAGAGTCAAAACTACCGGAACCTCCGATGGT‑CAGTGTGGATTCGAGCCTGATGGTCGAGCCGAACTTGACGACCGAGATGCTAAACGTATTCTCGCAGTTACCCAGAAGGGTGACGCCTGTATTCGTTCGTTACAGGATACTATTCGTGAACTGCAACTTAAGTAGGAAATCAAGTAAGGAGGCAATGTGTCTACTcctgtctcttatac                                                                                                                                                                               >  1:41895/1‑288 (MQ=255)`  
                                                                                                                                `cTGCGAAGTATCAAGAAGACCTTGCCACGCTGGAAGGGAGCACTGATAGGATTATTTCTGATTTGCGTAGCGACAATAAGCGGTTGCGCGTCAGAGTCAAAACTACCGGAACCTCCGATGGT‑CAGTGTGGATTCGAGCCTGATGGTCGAGCCGAACTTGACGACCGAGATGCTAAACGTATTCTCGCAGTGACCCAGAAGGGTGACGCATGGATTCGTGCGTTACAGGATACTATTCGTGAACTGCAACGTAAGTAGGAAATCAAGTAAGGAGGCAATGTGTCTACTAAATCCAATCGTaa                                                                                                                                                                               <  1:317118/301‑1 (MQ=255)`  
                                                                                                                                `aagacatgTATCAAGAAGACCTTGCCGAGCTGGAAGGGAGCACTGATAGGCTTATTTCGTATTTGCGTAGCGACAATAAGCGGTTGCGCGTCAGAGTCAAAACTACCGGAACCTCCGATGGT‑CAGTGTGTATTCGAGCCTGATGGTCGAGCCGAACTTGACGACCGAGATGCTAAACGTATTCTCGCAGTGACCCAGAAGGGTGACTCATGGATTCGTGCGTTACAGGTTACTATTCGTGAACTGCAACGTAAGTAGGAAATCAAGTAAGGAGGCAATGTGTCTACTCAATCCAATCGTaa                                                                                                                                                                               <  1:321351/294‑1 (MQ=255)`  
                                                                                                                                       `gTATCAAGAAGACCTTGCCGCGCTGGAAGGGAGCACTGATAGGCTTATTTCTGATTTGCGTAGCGACAATAAGCGGTTGCGCGACAGAGTCAAAACTACCGGAACCTCCGATGGT‑CAGTGTGGATTCGAGCCTGATGGTCGAGCCGAACTTGACGACCGAGATGCTAAACGTATTCTCGCAGTGACACAGAGGGGTGACGCATGGATTCGTGCGTTACAGAATACTATTCGTGAACTGCAACGTAAGTAGGAAATCAAGTGAGGAGGCAATGTGTCTACTCAATCCAATAGTaaatgacac                                                                                                                                                                        >  1:249378/1‑294 (MQ=255)`  
                                                                                                                                       `gTATCAAGAAGACCTTGCCGCGCTGGAAGGGAGCACTGATAGGATTATTTCTGATTTGCGTAGCGACAATAAGCGGTTGCGCGTCAGAGTCAAAACTACCGGAACCTCCGATGGT‑CAGTGTGGATTCGAGCCTGATGGTCGAGCCGAACTTGACGACCGAGATGCTAAACGTATTCTCGCAGTGACCCAGAAGGGTGACGCATGGATTCGTGCGTTACAGGATACTATTCGTGAACTGCAACGTAAGTAGGAAATCAAGTAAGGAGGCAATGTGTCTACTCAATCCAATCGTAATGCGCTc                                                                                                                                                                        >  1:265165/1‑301 (MQ=255)`  
                                                                                                                                       `gTATCAAGAAGACCTTGCCGCGCTGGAAGGGAGCACTGATAGGATTATTTCTGATTTGCGTAGCGACAATAAGCGGTTGCGCGTCAGAGTCAAAACTACCGGAACCTCCGATGGT‑CAGTGTGGATTCGAGCCTGATGGTCGAGCCGAACTTGACGACCGAGATGCTAAACGTATTCTCGCAGTGACCCAGAAGGGTGACGCATGGATTCGGGCGTTACAGGATACTATTCGTGAACTGCAACGTAAGTAGGAAATCAAGTAAGGAGGCAATGTGTCTACTCAATCCAATCGTAATGTGCTc                                                                                                                                                                        >  1:287858/1‑301 (MQ=255)`  
                                                                                                                                            `aagaagACCTTGCCGCGCTGGAAGGGAGCACTGATAGGATTATTTCTGATTTGCGTAGCGACAATAAGCGGTTGCGCGTCAGAGTCAAAACTACCGGAACCTCCGATGGT‑CAGTGTGGATTCGAGCCTGATGGTCGAACCGAACTTGACGACCGAGATGCTAAACGTATTCTCGCAGTGACCCAGAAGGGTGACGCATGGATTCGTGCGTTACAGGATACTATTCGTGAACTGCAACGTAAGTAGGAAATCAAGTAAGGAGGCAATGTGTCTACTCAATCCAATCGTAATgctgtctctta                                                                                                                                                                   >  1:241567/1‑292 (MQ=255)`  
                                                                                                                                                 `gaCCTTGCCGCGCTGGAAGGGAGCACTGATAGGATTATTTCTGATTTGCGTAGCGACAATAAGCGGTTGCGCGTCAGAGTCAAAACTACCGGAACCTCCGATGGT‑CAGTGTGGATTCGAGCCTGATGGTCGAGCCGAACTTGACGACCGAGATGCTAAACGTATTCTCGCAGTGACCCAGAAGGGTGGCGCATGGATTCGTGCGTTACAGGATACTATTCGTGAACTGCAACGTAAGTAGGAAATCAAGTAAGGAGGCAATGTGTCTACTCAATCCAATCGTAATGCGCTCGTAGTGgcgc                                                                                                                                                              >  1:74482/1‑301 (MQ=255)`  
                                                                                                                                                      `tGCCGCGCTGGAAGCGGGCACTGATAGGATTATTTCTGATTTGCGTAGCGACAATAAGCGGTTGCGCGTCAGAGTCAAAACTACCGGAACCTCCGATGGT‑CAGTGTGGATTCGAGCCTGATGGTCGAGCCGAACTTGACGACCGAGATGCTAAACGTATTCTCGCAGTGACCCAGAAGGGTGACGCATGGATTCGTGCGTTACAGGATACTATTCGTGAACTGCAACGTAAGCAGGAAATCAAGTAAGGAGGCAATGTGTCTACTCAATCCAATCGTAATGCGCTCGTAGTGGAGCAACTg                                                                                                                                                         <  1:439652/301‑1 (MQ=255)`  
                                                                                                                                                           `gggtGGAGGGGAGAAGGGATAGGGAGAGTTCGGATGTGCGAAGCGACAATAAGCGGTGGCGAGTCAGAGTCAAAACTACCGGAACCTCCGATGGT‑CAGTGTGGATTCGAGCCTGATGGTCGAGCCGAACTTGACGAACGAGATGCTAAACGTATTCTCGCAGTGACCCAGAAGGGTGAAGCATGGATTCGTGCGTTACAGGATACTATTCGTGAACTGCAACGTAAGTAGGAAATCAAGTAAGGAGGCAATGTGTCTACTCAATCCAATCGTAATGCGCTCGTAGTGGCGCAACTGAAAgg                                                                                                                                                    <  1:486792/298‑1 (MQ=255)`  
                                                                                                                                                           `ggcgggAAGGGAGCACTGATAGGATTATTTCTGATTGGCGTAGCGACAATAAGCGGTTGCGCGTCAGAGTAAAAACTACCGGAACCTCCGATGGT‑CAGTGTGGATTCGAGCCTGATGGTCGAGCCGAACTTGACGACCGAGATGCTAAACGTATTCTCGCAGTGACCCAGAAGGGTGACGCATGGATTCGTGCGTTACAGGATACTATTCGTGAACTGCAACGTAAGCAGGAAATCAAGTAAGGAGGCAATGTGTCTACTCAATCCAATCGTAATGCGCTCGTAGTGGCGCAACTGAAAgg                                                                                                                                                    <  1:273851/297‑1 (MQ=255)`  
                                                                                                                                                           `ggcTGGAAGGGAGCACTGATAGGATTATTTCTGATTTGCGTAGCGACAATAAGCGGTTGCGCGTCAGAGTCAAAACTACCGGAGCCTCCGATGGT‑CAGTGTGGATTCGAGCCTGATGGTCGAGCCGAACTTGACGACCGAGATGCTAAACGTATTCTCGCAGTGACCCAGAAGGGTGACGCATGGATTCGTGCGTTACAGGATACTATTCGTGAACTGCAACGTAAGTAGGAAATCAAGTAAGGAGGCAATGTGTCTACTCAATCCGATCGTAATGCGCTCGTAGTGGCGCAACTGAAAgg                                                                                                                                                    <  1:24647/300‑1 (MQ=255)`  
                                                                                                                                                             `cTGGAAGGGAGCACTGATAGGATTATTTCTGATTTGCGTAGCGACAATAAGCGGTTGCGCGTCAGAGTCAAAACTACCGGAACCTCCGATGGT‑CAGTGTGGATTCTAGCCTGATGGTCGAGCCGAACTTGACGACCGAGATGCTAAACGTATTCTCGCAGTGACCCAGAAGGGTGACGCATGGATTCGTGCGTTACAGGATACTATTCGTGAAATGCAACGTAAGTAGGAAATCAAGTAAGGAGGCACAGTGTCTAATCACTCCAATCGTAATGCGCTCGTAGTGGCGCAACTGAAAGgag                                                                                                                                                  >  1:44457/1‑301 (MQ=255)`  
                                                                                                                                                                          `cTGATAGGATTATTTCTGATTTGCGTAGCGACAATAAGCGGTTGCGCGTCAGAGTCAAAACTACCGGAACCTCCGATGGT‑CAGTGTGGATTCGAGCCTGATGGTCGAGCCGAACTTGACGACCGAGATGCTAAACGTATTCTCGCAGTGACCCAGAAGGGTGACGCATGGATTCGTGCGTTACAGGATACTATTCGTGAACTGCAACGTAAGCAGGAAATCAAGTAAGGAGGCAATGTGTCTACTCAATCCAATCGTAATGCGCTCGTAGTGGCGCAACTGAAAGGAGACTTCGTGGCGtt                                                                                                                                     >  1:151058/1‑301 (MQ=255)`  
                                                                                                                                                                            `gATAGGATTATTTCTGATTTGCGTAGCGACAATAAGCGGTTGCGCGTCAGAGTCAAAACTACCGGAACCTCCGATGGT‑CAGTGTGGATTCGAGCCTGATGGTCGAGCCGAACTTGACGACCGAGATGCTAAACGTATTCTCGCAGTGACCCAGAAGGGTGACGCATGGATTCGTGCGTTATAGGATACTATTCGTGAACTGCAACGTAAGTAGGAAATCAAGTAAGGAGGCAATGTGTCTACTCAATCCAATCGTAATGCGCTCGTAGTGGCGCAACTGAAAGGAGACTTCCTgtctcttt                                                                                                                                   >  1:171355/1‑294 (MQ=255)`  
                                                                                                                                                                              `tAGGATTATTTCTGATTTGCGTAGCGACAATAAGCGGTTGCGCGTCAGAGTCAAAACTACCGGAACCTCCGATGGT‑CAGTGTGGATTCGAGCCTGATGGTCGAGCCGAACTTGACGACCGAGATGCTAAACGTATTCTCGCAGTGACCCAGAAGGGTGACGCATGGATTCGTGCGTTACAGGATACTATTCGTGAACTGCAACGTAAGTAGGAAATCAAGTAAGGAGGCAATGTGTCTACTCAATCCAATCGTAATGCGCTCGTAGTGGCGCAACTGAAAGGAGACTTCGTGGCGTTCCTa                                                                                                                                 <  1:413828/301‑1 (MQ=255)`  
                                                                                                                                                                              `agagacagtttCTGATTTGCGTAGCGACAATAATCGGTTGCGCGTCAGAGTCAAAACTACCGGAACCTCCGATGGT‑CAGTGTGGATTCGAGCCTGATGGTCGAGCCGAACTTGACGACCGAGATGCTAAACGTATTCTCGCAGTGACCCAGAAGGGTGACGCATGGATTCGTGCGTTACAGGATACTATTCGTGAACTGCAACGTAAGTAGGAAATCAAGTAAGGAGGCAATGTGTCTACTCAATCCAATCGTAATGCGCTCGTAGTGGCGCAACTGAAAGGAGACTTCGTGGCGTTACTa                                                                                                                                 <  1:3947/293‑1 (MQ=255)`  
                                                                                                                                                                                `gtattattTCTGATTTGCGTAGCGACAATAAGCGGTTGCGCGTAAGAGTCAAAACTACCGGAACCTCCGATGGT‑CAGTGTGGATTCGAGCCTGATGGTCGAGCCGAACTTGACGATCGAGATGCTAAACGTATTCTCGCAGTGACCCAGAAGGGTGACGCATTGATTCGTGCGTTACAGGATACTATTCGTGAACTGCAACGTAAGTAGGAAATCAAGTAAGGAGGCAATGTGTCTACTCAATCCAATCGTAATGCGCTCGTAGTGGCGCAACTGAAAGGAGACTTCGTGGCGTTCCTAtt                                                                                                                               >  1:117610/3‑301 (MQ=255)`  
                                                                                                                                                                                   `cagtttCTGAATTGCGTAGCGACAATAAGCGGTTGCGCGTCAGAGTCAAAACTACCGGAACCTCCGATGGT‑CAGTGTGGATTCGAGCCTGATGGTCGAGCCGAACTTGACGACCGAGATGCTAAACGTATTCTCGCAGTGACCCAGAAGGGTGACGCATGGGTTCGTGCGTTACAGGATACTATTCGTGAACTGCAACGTAAGTAGGAAATCAAGTAAGGAGGCAATGTGTCTACTCAATCCAATCGTAATGCGCTCGTAGTGGCGCAACTGAAAGGAGACTTCGTGGCGTTCCTATTCGt                                                                                                                            <  1:23644/298‑1 (MQ=255)`  
                                                                                                                                                                                    `tatcTCTGATTTGCGTAGTGTCAATAAGCGATTGCGCGTCAGAGTCAAAACTACCGGAACCTCCGATGGT‑CAGTGTGGATTCGAGCATGATGGTCGAGCCGAACTTGACGACCGAGATGCTAAACGTCTTCTCGCAGTGACCCAGAAGGGTGACGCATGGATTCGTGCGTTACAGGATACTATTCGTTAACTGCAACGTAAGTAGGAAATCAAGTAAGGAGGCAATGTGTCTACTCAATCCAATCGTAATGCGCTCGTAGTGGCGCAACTGAAAGGAGACTTCGTGGCGTTCCTATTCGTc                                                                                                                           <  1:503380/301‑1 (MQ=255)`  
                                                                                                                                                                                      `tttCTGATTTGCGTAGCGACAATAAGCGGTTGCGCGTCAGAGTCAAAACTACCGGAACCTCCGATGGT‑CAGTGTGGATTCGAGCCTGATGGTCGAGCCGAACTTGACGACCGAGATGCTAAACGTATTCTCGCAGTGACCCAGAAGGGTGACGCATGGATTCGTGCGTTACAGGATACTATTCGTGAACTGCAACGTAAGTAGGAAATCAAGTAAGGAGGCAATGTGTCTACTCAATCCAATCGTAATGCGCTCGTAGTGGCGCAACTGAAAGGAGACTTCGTGGCGTTCCTATTCGTCtt                                                                                                                         >  1:12521/1‑301 (MQ=255)`  
                                                                                                                                                                                          `ttaTTTGCGTAGCGACAATAAGCGGTTGCGCGTCAGAGTCAAAACTACCGGAACCTCCGATGGT‑CAGTGTGGATTCGAGCCTGATGGTCGAGCCGAACTTGACGACCGAGATGCTAAACGTATTCTCGCAGTGACCCAGAAGGGTGACGCATGGATTCGTGCGTTACAGGATACTATTCGTGAACTGCAACGTAAGTAGGAAATCAAGTAAGGAGGCAATGTGTCTACTCAATCCAATCGTAATGCGCTCGTAGTGGCGCAACTGAAAGGAGACTTCGTGGCGTTCCTATTCGTCTTATgg                                                                                                                     <  1:430251/299‑1 (MQ=255)`  
                                                                                                                                                                                          `agATTGGCGTGGAGACAAAAAGCGGTTGCGCGTCAGAGTCAAAACTACCGGAACCTCCGATGGT‑CAGGGTGGATTCGAGCCTGATGGTCGAGCCGAACTTGACGAACGAGGTGCTAAACGTATTCTCGCAGTGACCAAGAAGGGTGACGCATGGATTCGTGCGTTACAGGATACTATTCGTGAACTGCAACGTAAGTAGGAAATCAAGTAAGGAGGCAATGTGTCTACTCAATCCAATCGTAATGCGCTCGTAGTGGCGCAACTGAAAGGAGACTTCGTGGCGTTCCTATTCGTCTTATgg                                                                                                                     <  1:134571/300‑1 (MQ=255)`  
                                                                                                                                                                                                `gCGTAGCGACAATAAGCGGTTGCGCGTCAGAGTCAAAACTACCGGAACCTCCGATGGT‑CAGTGTGGATTCGAGCCTGATGGTCGAGCCGAACTTGACGACCGCGATGCTAAACGTATTCTCGCAGTGACCCAGAAGGGTGACGCATGGATTCGTGCGTTACAGGATACTATTCGTGAACTGCAACGTACGTAGGACATCAAGTAAGGATGCCATGTGTCTACTCAATCCACTCGTAATGCGCTCGTATTGACACAACTGAAAGGAGACTTCGTGGCGTTCCTATTCGTCTTATGGAAGgcg                                                                                                               >  1:45423/1‑301 (MQ=255)`  
                                                                                                                                                                                                 `cGTAGCGACAATAAGCGGTTGCGCGGCAGAGTCAAAACTACCGGAACCTCCGATGGT‑CAGTGTGGATTCGAGCCTGATGGTCGAGCCGAACTTGACGACCGAGATGCTAAACGTATTCTCGCAGTGACCCAGAAGGGTGACGCATGGATTCGTGCGTTACAGGATACTATTCGTGAACTGCAACGTAAGTAGGAAATCAAGTAAGGAGGCAATGTGTCTACTCAATCCAATCGTAATGCGCTCGTAGTGGCGCAACTGAAAGGAGACTTCGTGGCGTTCCTATTCGTCTTATGGAAGgcgc                                                                                                              <  1:224619/301‑1 (MQ=255)`  
                                                                                                                                                                                                      `gacagaTAAGAGGTTGCGCGTCAGATTCAAAACTACCGGACTCTCCGATGGT‑CAGTGTGGAGTCGAGCCTGAGGGTCGAGCCGAACTTGACGACAGAGATGCTAAACGTATTCTCGTAGTGACCCAGAAGGGTGACGCATGGATACGTGCGTTACAGGATACTATTCGTGAACTGCAACGTAAGTAGGAAATCAAGTAAGGAGGCAATGTGTCTAATCAATCCAATCGTAATGCGCTCGTAGTGGCGCAACTGAAAGGAGACTTCGTGGCGTTCCTATTCGTCTTATGGAAGGCGCTAAAc                                                                                                         <  1:46842/296‑1 (MQ=255)`  
                                                                                                                                                                                                           `aTAAGCGGTTGCGCGTCAGAGTCAAAACTACCGGAACCTCCGATGGT‑CAGTGTGGATTCGAGCCTGATGGTCGAGCCGAACTTGACGACCGAGATGCTAAACGTATTCTCGCAGTGACCCAGAAGGGTGACGCATGGATTCGTGCGTTACAGGATACTATTCGTGAACTGCAACGTAAGTAGGAAATCAAGTAAGGAGGCAATGTGTCTACTCAATCCAATCGTAATGCGCTCGTAGTGGCGCAACTGAAAGGAGACTTCGTGGCGTTCCTATTCGTCTTATGGAAGTCGCTCAACCTAcc                                                                                                    >  1:326959/1‑301 (MQ=255)`  
                                                                                                                                                                                                             `aaGCGGTTGCGCGTCAGAGTCAAAACTACCGGAACCTCCGATGGT‑CAGTGTGGATTCGAGCCTGATGGTCGAGCCGAACTTGACGACCGAGATGCTAAACGTATTCTCGCAGTGACCCAGAAGGGTGACGCATGGATTCGTGCGTTACAGGATACTATTCGTGAACTGCAACGTAAGTAGGAAATCAAGTAAGGAGGCAATGTGTCTACTCAATCCAATCGTAATGCGCTCGTAGTGGCGCAACTGAAAGGAGACTTCGTGGCGTTCCTATTCGTCTTATGGAAGGCGCTAAACCTACCgg                                                                                                  <  1:477939/301‑1 (MQ=255)`  
                                                                                                                                                                                                                        `tgtatcagaggaagatACCGGAACCGCCGATGGT‑CAGTGGGGAGTCGAGCCTGATGGTCGAGCCGAACTTGACGACCGAGATGCTAAACGTATTCTCGCAGTGACCCAGAAGGGTGACGCATGGATTCGTGCGTTACAGGATACTAGTCGTGAACTGCAACGTAAGTAGGAAATCAAGTAAGGAGGCAATGTGTCTACTCAATCCAATCGTAATGCGCTCGTAGTGGCGCAACTGAAAGGAGACTTCGTGGCGTTCCTATTCGTCTTATGGAAGGCGCTAAACCTACCGGTGCCCACTAAg                                                                                       <  1:410000/286‑1 (MQ=255)`  
                                                                                                                                                                                                                                `ccAAAGCTACTGAAACCTTC‑ATGGTGCAGTGTTGATTCGGGCCTGATGGACGAGCCGGACTTGGCGACCGGGATGCTAAACGTATTCTCGGAGTGACCCAGAAGGGTGACGCATGGATTTGGGCGTTACAGGATACTATTCGTGAACTGCAACGTAAGTAGGAAATCAATTAAGGAAGCAATGTGTCTACTCAATCCAATCGTGATGCGCTCGTAGTGGAGCAACTGATAGGAGACTTCGTGGCGTTCCTATTCGTCTTATGGAAGGCGCTAAACCTACCGGTGCCCACTAAGTGTCAGAt                                                                               <  1:253043/300‑1 (MQ=255)`  
                                                                                                                                                                                                                                    `aaCTACCGGAACCTCCGATGGT‑CAGTGTGGATTCGAGCCTGATGGTCGAGCCGAACTTGACGACCGAGATGCTAAACGTATTCTCGCAGTGACCCAGAAGGGTGACGCATGGATTCGTGCGTTACAGGATACTATTCGTGAACTGCAACGTAAGTAGGAAATCAAGTAAGGAGGCAATGTGTCTACTCAATCCAATCGTAATGCGCTCGTAGTGGCGCAACTGAAAGGAGACTTCGTGGCGTTCCTATTCGTCTTATGGAAGGCGCTAAACCTACCGGTGCCCACTAAGTGTCAGATTGAc                                                                           <  1:142855/301‑1 (MQ=255)`  
                                                                                                                                                                                                                                       `tACCGGAACCTCCGATGGT‑CAGTGTGGATTCGAGCCTGATGGTCGAGCCGAACTTGACGACCGAGATGCTAAACGTATTCTCGCAGTGACCCAGAAGGGTGACGCATGGATTCGTGCGTTACAGGATACTATTCGTGAACTGCAACGTAAGTAGGAAATCAAGTAAGGAGGCAATGTGTCTACTCAATCCAATCGTAATGCGCTCGTAGTGGAGCAACTGAAAGGAGACTTCGTGGCGTTCCTATTCGTCTTATGGAAGGCGCTAAACCTACCGGTGCCCACTAAGTGTCAGATTGACATg                                                                        >  1:103801/1‑301 (MQ=255)`  
                                                                                                                                                                                                                                          `cgaagccTCCGAGGGT‑CAGTGTGGATTCGAGTCTGAGGGTCGAGCAGAAGTTGACTACCGAGATGGTAAACGTAGTCTCTCAGGGACCCAGAAGGGTGACGCATGGATTCGTGCGTTACAGGATACTATTCGTGAACTGCAACGTAAGTAGGAAATAAAGTAAGGAGGCAATGTGTCTACTAAATCCAATCGTAATGCGCTCGTAGTGGCGCAACTGAAAGGAGACTTCGTGGCGTTCCTATTCGTCTGATGGAAGGCGCTAAACCTACCGGTGCCCACTAAGTGTCAGATTGACATGGCt                                                                     <  1:449412/296‑1 (MQ=255)`  
                                                                                                                                                                                                                                          `cGGAACCTCCGAGGGT‑CAGTGTGGATTCGAGCCTGATGGTCGAGCCGAACTTGACGAACGAGATGCTAAACGTATTCTCGCAGTGACCCAGAAGGGTGACGCATGGATTCGTGCGTTACAGGATACTATTCGTGAACTGCAACGTAAGCAGGAAATCAAGTAAGGAGGCAATGTGTCTACTCAATCCAATCGTAATGCGCTCGTAGTGGCGCAACTGAAAGGAGACTTCGTGGCGTTCCTATTCGTCTTATGGAAGGCGCTAAACCTACCGGTGCCCACTAAGTGTCAGATTGACATGGCt                                                                     <  1:359387/301‑1 (MQ=255)`  
                                                                                                                                                                                                                                               `ccTCCGATGGT‑CAGTGTGGATTCGAGCCTGATGGTCGAGCCGAACTTGACGACCGAGATGCTAAACGTATTCTCGCAGTGACCCAGAAGGGTGACGCATGGATTCGTGCGTTACAGGATACTATTCGTGAACTGCAACGTAAGTAGGAAATCAAGTAAGGAGGCAATGTGTCTACTCAATCCAATCGTAATGCGCTCGTAGTAGCGCAACTGAAAGGAGACTTCGTGGCGTTCCTATTCGTCTTATGGAAGGCGCTAAACCTACCGGTGCCCACTAAGTGTCAGATTGACATGGCTAAGGt                                                                >  1:230100/1‑301 (MQ=255)`  
                                                                                                                                                                                                                                                  `ccGATGGT‑CAGTGTGGATTCGAGCCTGATGGTCGAGCCGAACTTGACGACCGAGATGCTAAACGTATTCTCGCAGTGACCCAGAAGGGTGACGCATGGATTCGTGCGTTACAGGATACTATTCGTGAACTGCAACGTAAGCAGGAAATCAAGTAAGGAGGCAATGTGTCTACTCAATCCAATCGTAATGCGCTCGTAGTGGCGCAACTGAAAGGAGACTTCGTGGCGTTCCTATTCGTCTTATGGAAGGCGCTAAACCTACCGGTGCCCACTAAGTGTCAGATTGACATGGCTAAGGTGCt                                                             >  1:203249/1‑301 (MQ=255)`  
                                                                                                                                                                                                                                                  `ccGATGGT‑AAGTGTGGATTCGAGCCTGATGGTCGAGCCGAACTTGACGACCGAGATGCTAAACGTATTCTCGCAGTGACCCAGAAGGGTGACGCATGGATTCGTGCGTTACAGGATACTATTCGTGAACTGCAACGTAAGTAGGAAATCAAGTAAGGAGGCAATGTGTCTACTCAATCCAATCGTAATGCGCTCGTAGTGGCGCAACTGAAAGGAGACTTCGTGGCGTTACTATTCGTCTTATGGAAGGCGCTAAACCTACCGGTGCCCACTAAGGGTCAGATTGACATGGCTAAggatgt                                                             >  1:434105/1‑297 (MQ=255)`  
                                                                                                                                                                                                                                                            `aGTGTGCATTCTACCCTGATAGTCGAGCCGAACTTGACGACCGTGATGCTAACCGTATTCTCGCAGTGACCCAGAAGAGTGACGCATGGATTCGTGCGTTACAGGATACTATTCGTGAACTGCAACGTAAGTAGGAAATCAAGTAAGGAGGCAATGTGTCTACTCAATCCAATCGTAATGCGCTCGTAGTGGCGCAACTGAAAGGAGACTTCGTGGCGTTCCTATTCGTCTTATGGAAGGCGCTAAACCTACCGGTGCCCACTAAGTGTCAGATTGACATGGCTAAGGTGCTGGCGAATgg                                                    <  1:166365/301‑1 (MQ=255)`  
                                                                                                                                                                                                                                                             `gtgtGGATTCGAGCCTGATGGTCGATCCGAACTTGACGACCGAGATGCTAAACGTATTCTCGCAGTGACCCAGAAGGGTGACGCATGGATTCGTGCGTTACAGGATACTATTCGTGAACTGCAACGTAAGTAGGAAATCAAGTAAGGAGGCAATGTGTCTACTCAATCCAATCGTAATGCGCTCGTAGTGGCGCAACTGAAAGGAGACTTCGTGGCGTTCCTATTCGTCTTATGGAAGGCGGTAAACCTACCGGTGCCCACTAAGTGGCAGATTGACATGGCTAAGGTGCTGGCGAATGga                                                   >  1:175612/1‑301 (MQ=255)`  
                                                                                                                                                                                                                                                             `gtgtGGATTCGAGCCTGATGGTCGAGCCGAACTTGACGACCGAGATGCTAAACGTATTCTCGCAGTGACCCAGAAGGGTGACGCATGGATTCGTGCGTTACAGGATACTATTCGTGAACTGCAACGTACGTAGGAAATCAAGTAAGGAGGCAATGTGTCTACTCAATCCAATCGTAATGCGCTCGTAGTGGCGCAACTGAAAGGAGACTTCGTGGCGTTCCTATTCGTCTTATGGAAGGCGCTAAACCTACCGGTGCCCACTAAGTGTCAGATTGACATGGCTAAGGTGCTGGCgctgtct                                                   >  1:59353/1‑295 (MQ=255)`  
                                                                                                                                                                                                                                                                                       `cctaaCTTGACGACCTAGATGCTAAACGGATGCTCGCAGTGACCCAGAAGGGTGCCGCATTGATTCGTTCGTTACAGGATACTATTCGTGAACTGCAACGTAAGTAGGAAATCAAGTAAGGATGCAATGTGTCTACTCAATCCAATCGTAATGCGCTCGTAGTGGCGCAACTGAAAGGAGACTTCGTGGCGTTCCTATTCGTCTTATGGAAGGCGCTAAACCTCCCGGTGCCCACTAAGTGTCAGATTTACATTGCTAAGGTGCTGGCTAATGGAGACAACAAGAAGTTCATCTTACAGGc                         <  1:509993/298‑1 (MQ=255)`  
                                                                                                                                                                                                                                                                                          `aaCTTGACGACCGAGATGCTAAACGTATTCTCGCAGTGACCCAGAAGGGTGACGCATGGATTCGTGCGTTACAGGATACTATTCGTGAACTGCAACGTAAGTAGGAAATCAAGTAAGGAGGCAATGTGTCTACTCAATCCAATCGTAATGCGCTCGTAGTGGCGCAACTGAAAGGAGACTTCGTGGCGTTCCTATTCGTCTTATGGAAGGCGCTAAACCTACCGGTGCCCACTAAGTGTCAGATTGACATGGCTAAGGAGCTGGCGAATGGAGACAACAAGAAGTTctgtctcttatacac                      >  1:283797/1‑287 (MQ=255)`  
                                                                                                                                                                                                                                                                                              `tGACGACCGAGATGCTAAACGTATTCTCGCAGTGACCCAGAATGGTGACGCATGGATTCGTGCGTTACAGGATACTATTCGTGAACTGCAACGTAAGTAGGAAATCAAGTAAGGAGGCAATGTGTCTACTCAATCCAATCGTAATGCGCTCGTAGTGGCGCAACTGAAAGGAGACTTCGTGGCGTTCCTATTCGTCTTCTGGAAGGCGCTACACCTACCGGTGCCCACTAAGTGTCAGATTGACATGGCTAAGGTGCTGGCGAATGGAGACAACAAGAAGTTCATCTTACAGGCTTTCCgc                  >  1:188962/1‑300 (MQ=255)`  
                                                                                                                                                                                                                                                                                                          `tGCTAAACGTATTCTCGCAGTGACCCAGAAGGGTGACGCATGGATTCGTGCGTTACAGGATACTATTCGTGAACTGCAACGTAAGTAGGAAATCAAGTAAGGAGGCAATGTGTCTACTCAATCCAATCGTAATGCGCTCGTAGTGGCGCAACTGAAAGGAGACTTCGTGGCGTTCCTATTCGTCTTATGGAAGGCGCTAAACCTACCGGTGCCCACTAAGTGTCAGATTGACATGGCTAAGGTGCTGGCGAATGGAGACAACAAGAAGTTCATCTTACAGGCTTTCCGTGGTATCGGTAAg      >  1:83634/1‑301 (MQ=255)`  
                                                                                                                                                                                                                                                                                                           `gCTAAACGTATTCTCGCAGTGACCCAGAAGGGTGACGCATGGATTCGTGCGTTACAGGATACTATTCGTGAACTGCAACGTAAGTAGGAAATCAAGTAAGGAGGCAATGTGTCTACTCAATCCAATCGTAATGCGCTCGTAGTGGCGCAACTGAAAGGAGACTTCGTGGCGTTCCTATTCGTCTTATGGAAGGCGCTAAACCTACCGGTGCCCACTAAGTGTCAGATTGACATGGCTAAGGTGCTGGCGAATGGAGACAACAAGAAGTTCATCTTACAGGCTTTCCGTGGTATCGGTCAGt     >  1:459273/1‑301 (MQ=255)`  

                                                                                                                                                                                                                                                                                                              `|`                                                                                                                                                                                                                                                                                                              
`GCTATTCGGGTTAGGATGGCATCTAGGGTCAGACTCAATGGACGCTAAATGGAAACAGGAGGTACACAATGAGTACGTTAAGAGAGTTGAGGCTGCGAAGAGCACTCAAAGAGCAATCGATGCGGTATCTGCTAAGTATCAAGAAGACCTTGCCGCGCTGGAAGGGAGCACTGATAGGATTATTTCTGATTTGCGTAGCGACAATAAGCGGTTGCGCGTCAGAGTCAAAACTACCGGAACCTCCGATGGT‑CAGTGTGGATTCGAGCCTGATGGTCGAGCCGAACTTGACGACCGAGATGCTAAACGTATTCTCGCAGTGACCCAGAAGGGTGACGCATGGATTCGTGCGTTACAGGATACTATTCGTGAACTGCAACGTAAGTAGGAAATCAAGTAAGGAGGCAATGTGTCTACTCAATCCAATCGTAATGCGCTCGTAGTGGCGCAACTGAAAGGAGACTTCGTGGCGTTCCTATTCGTCTTATGGAAGGCGCTAAACCTACCGGTGCCCACTAAGTGTCAGATTGACATGGCTAAGGTGCTGGCGAATGGAGACAACAAGAAGTTCATCTTACAGGCTTTCCGTGGTATCGGTAAGTCGT  >  NC_001604.1/36964‑37565`

  

Alignment Legend

Aligned base mismatch/match (shaded by quality score): `ATCG/ATCG` `< 3 ≤` `ATCG/ATCG` `< 9 ≤` `ATCG/ATCG` `< 20 ≤` `ATCG/ATCG` `< 34 ≤` `ATCG/ATCG` `< 38 ≤` `ATCG/ATCG`

Unaligned base: `atcg`    Masked matching base: `atcg`    Alignment gap: `‑`    Deleted base: `‑`

Reads not counted as support for junction

read\_name Not counted due to insufficient overlap past the breakpoint.

read\_name Not counted due to not crossing MOB target site duplication.
